# Is polycystic ovary syndrome a risk factor for depression and anxiety?: a cross-sectional study

**DOI:** 10.1590/1806-9282.20230918

**Published:** 2024-04-22

**Authors:** Osman Samet Gunkaya, Arzu Bilge Tekin, Ayşegül Bestel, Oguz Arslan, Fatih Şahin, Bilge Dogan Taymur, Niyazi Tuğ

**Affiliations:** 1 University of Health Sciences Turkey, Sehit Prof. Dr. Ilhan Varank Sancaktepe Training and Research Hospital, Department of Obstetrics and Gynecology – İstanbul, Turkey.; 2 University of Health Sciences Turkey, Kanuni Sultan Suleyman Training and Research Hospital, Clinic of Obstetrics and Gynecology – İstanbul, Turkey.; 3 University of Health Sciences Turkey, Istanbul Prof. Dr. Cemil Taşcioğlu City Hospital, Department of Obstetrics and Gynecology – İstanbul, Turkey.

**Keywords:** Depression, Anxiety, Polycystic ovary syndrome, Hyperandrogenism

## Abstract

**OBJECTIVE:**

The objective of this study was to learn more about the prevalence and pathophysiology of depression and anxiety that may be caused by polycystic ovary syndrome and to make plans for taking necessary precautions for this vulnerable group.

**METHODS:**

This case-control study was conducted between January 2022 and October 2022. A total of 120 women with polycystic ovary syndrome and 143 controls were included in the study. All healthy volunteers and women with polycystic ovary syndrome were evaluated using self-administered questionnaires and physical examination. Anthropometric data such as weight and height and laboratory value were documented.

**RESULTS:**

There was no significant difference between the groups in terms of demographic characteristics. When the Hospital Anxiety and Depression Scale scores of both groups were compared, both depression and anxiety scores were found to be significantly higher in women with polycystic ovary syndrome compared with the control group (OR: 3.319, 95%CI, 1.563–7.047, p<0.001 and OR: 3.238, 95%CI, 1.659–6.315, p<0.001). In the Hospital Anxiety and Depression Scale questionnaire, the rate of irregular menstruation and Ferriman-Gallwey score were statistically significant in women with polycystic ovary syndrome with high depression and anxiety scores. While serum LH levels and LH/FSH ratios were significantly different in women with polycystic ovary syndrome with high depression scores, serum LH, LH:FSH ratios, and serum total testosterone levels were found significant in women with polycystic ovary syndrome with high anxiety scores.

**CONCLUSION:**

It is clear that depression and anxiety are more common in patients with polycystic ovary syndrome than in healthy women. Our findings support previous recommendations regarding routine screening for depression and anxiety in this population.

## INTRODUCTION

Polycystic ovary syndrome (PCOS) is a chronic gynecological disease with endocrine and metabolic components that affects approximately 5–20% of women of childbearing age^1^. The characteristic clinical features are menstrual irregularities, hirsutism, polycystic ovaries on ultrasound, and clinical or laboratory findings of androgen increase^1,2^. Women with PCOS are at risk for metabolic, cardiovascular, and many systemic diseases (diabetes, dyslipidemia, hypertension, metabolic syndrome, obstructive sleep apnea, pregnancy complications, and endometrial cancer), especially obesity^3^. Although these endocrine and metabolic results increase the incidence of psychiatric disorders such as depression and anxiety in women with PCOS, the underlying mechanisms are not fully understood^4,5^. Chronic illness is a known risk factor for depression, but less is known about the interaction between chronic illness and anxiety^6^. Additionally, although a significant link between PCOS and sexual dysfunction has not been proven, some studies have reported that most women with PCOS experience more stress than healthy women due to infertility concerns and dissatisfaction with their body image^7,8^. Furthermore, whether treated or not, these women's phenotype and clinical course are of great concern^9^.

Androgen excess is found in almost 85% of women with PCOS, indicating that this syndrome is the main cause of hirsutism^10,11^. It has been observed that women with PCOS who experience emotional distress exhibit clinical symptoms of alopecia, acne, hirsutism, and infertility^12^. Moreover, the neuroendocrine feature of PCOS is sustained rapid LH (GnRH) pulsatility; this increases the pituitary synthesis of LH over that of FSH, resulting in increased LH concentrations and increased PCOS-specific LH:FSH ratios. Low levels of FSH contribute to ovulatory dysfunction, while high levels of LH increase ovarian androgen production^13^. Increased blood testosterone levels have also been associated as a biochemical factor in increased depression and anxiety in PCOS^13^.

The aim of this study was to learn more about the prevalence of depression and anxiety that can be caused by PCOS and the impact of physical and biochemical changes on depression and anxiety in PCOS women and to make plans to take the necessary precautions for this sensitive group.

## METHODS

We conducted this case-control study on volunteers who applied to the gynecology department at a tertiary medical center between January 2022 and October 2022. The study was conducted with the institutional ethics committee (E-46059653-020). It complies with the Declaration of Helsinki. All patients confirmed written informed consent before they were enrolled in the study. The diagnosis of PCOS was made using the Rotterdam criteria^2^. Briefly, the diagnosis of PCOS required hyperandrogenic symptoms (hirsutism, acne, and androgenic alopecia) and oligomenorrhea (menstrual cycle more than 35 days apart)/amenorrhea (absence of menstrual cycle for at least 6 months), as well as biochemical hyperandrogenemia (serum total testosterone level.0.77 ng/mL) and polycystic ovaries (12 follicles [2–9 mm in size] per ovary by transvaginal ultrasonography or an ovarian volume >10 mL per ovary by transabdominal ultrasound with distended bladder for virgin women)^2^. The Ferriman-Gallwey grading system was used to assess the presence of hirsutism^10^. None of the women have been prescribed medication for symptoms prior to enrollment. As a control group, the healthy volunteers, not diagnosed with PCOS, who came to the same facility for routine health check-ups, were invited to participate in this study. Exclusion criteria were treatment of depression and anxiety, other endocrine, systemic abnormalities, and use of oral contraceptives and drugs known to affect the hypothalamic-pituitary-ovarian system. All healthy volunteers and women with PCOS were evaluated using self-administered questionnaires (Hospital Anxiety and Depression Scale (HADS)), face-to-face interviews with a gynecologist, and physical examination. All participants in the control group had regular ovulatory cycles (mean cycle length 25–32 days) and showed no clinical or biochemical signs of hyperandrogenism. Anthropometric data such as weight and height were measured by a qualified nurse. To evaluate laboratory findings, blood samples were taken from women with PCOS on the third day of the spontaneous or induced menstrual cycle, in the early follicular phase.

The HADS questionnaire was used to measure depression and anxiety. The HADS is a validated questionnaire consisting of 14 questions, with 7 questions to assess anxiety and 7 questions to assess depressive symptoms. The HADS score of 11 or higher was considered abnormal^14^. The women with high HADS scores were referred to the psychiatry department.

Statistical analysis was performed using SPSS, version 21. The conformity of the variables to the normal distribution was evaluated with the Shapiro-Wilk test, Q-Q plot, and histogram graphics. Continuous variables were presented as mean±standard deviation, median (quartiles 25–75), or (minimum–maximum). Categorical data were presented as frequency (percentage). For analyzing continuous data, the Mann-Whitney U test was used. Analyzing categorical variables, the chi-square test was employed. The significance level was defined as a p-value less than 0.05.

## RESULTS

**Figure 1:**
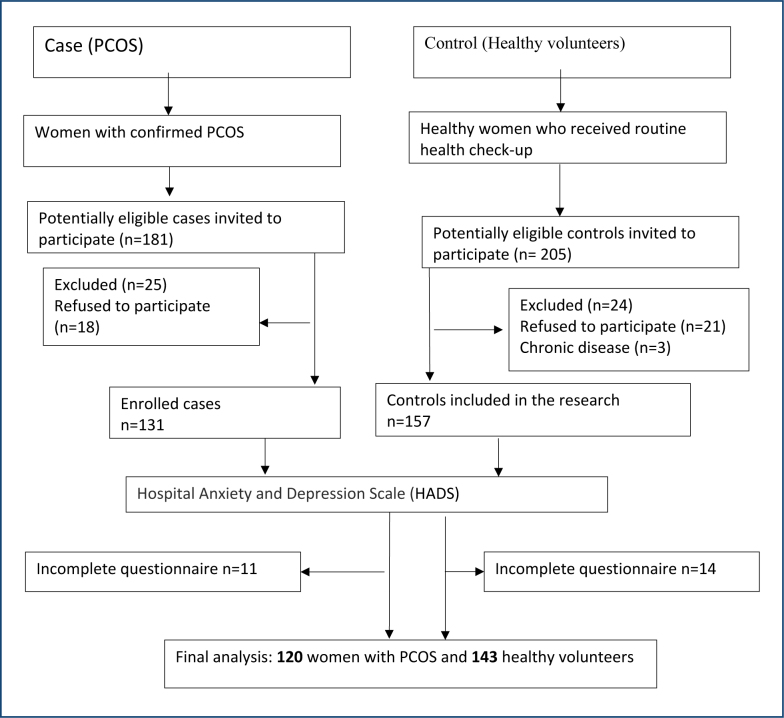
Flow diagram.

A total of 120 women with PCOS and 143 controls were included in the research. The flow diagram is shown in Figure 1. In this study, the prevalence of anxiety and depression behaviors in women with PCOS was 27.5 and 21.7%, respectively. When women with PCOS were evaluated within themselves, although there was no difference between the groups in terms of BMI, menstrual irregularity and high Ferimann-Gallwey scores were related to high depression and anxiety scores. In addition, serum LH levels, LH:FSH ratios, and serum total testosterone levels were found to be statistically significant in women with PCOS diagnosis with high anxiety scores.

There was no significant difference between women with PCOS and the control group in terms of demographic characteristics (Table 1). When the HADS scores of both groups were compared, both depression and anxiety scores were found to be significantly higher in women with PCOS compared with the control group (Table 1) OR: 3.319, 95%CI, 1.563–7.047, p<0.001 and OR: 3.238, 95%CI, 1.659–6.315, p<0.001, respectively. Women with PCOS who had a high depression score (HADS≥11) in the HADS questionnaire had a statistically significant difference in the rate of irregular menstruation (76.9%, n=20, p< 0.001) and Ferriman-Gallwey scores (9.5±6.0 median±IQR, p=0.014) when compared with those with low depression scores in terms of clinical features. Other clinical parameters are detailed in Table 2. The statistical comparison of women with PCOS with a high depression score (HADS≥11) and women with PCOS with a low depression score in terms of laboratory results is shown in Table 2. Women with PCOS who had higher anxiety scores on the HADS questionnaire (HADS≥11) had statistically significant differences in menstrual irregularity rate (84.8%, n=28, p<0.001) and Ferriman-Gallwey score 9.0 (6.0; median (IQR), p<0.001). There was a difference compared with those with low scores for clinical features of anxiety. Other clinical parameters are detailed in Table 3. When women with PCOS with high anxiety scores (HADS≥11) and women with PCOS with low anxiety scores were compared statistically in terms of laboratory results, serum LH, LH:FSH ratio, and serum total testosterone levels were found to be significant (Table 3).

**Table 1:** Demographic characteristics and Hospital Anxiety and Depression Scale of women with polycystic ovary syndrome and controls.

		PCOS (n=120)	Controls (n=143)	p		
Age (years) mean±SD		26.1±5.8 (16–44)	25.1±5.8 (16–44)	0.173^1^		
BMI (kg/m^2^) mean±SD		26.4±4.9 (17.4–45.8)	26.0±4.3 (17.3–33.8)	0.435^1^		
Gravity median (IQR)		0 (1) (0–6)	0 (1) (0–6)	0.989^2^		
Parity median (IQR)		0 (1) (0–4)	0 (1) (0–4)			
**Type of delivery**				0.871^2^		
	Nulliparty, n (%)	78 (65)	91 (63.6)			
	Vaginal birth, n (%)	23 (19.2)	31 (21.7)			
	Cesarean, n (%)	19 (15.8)	21 (14.7)			
HADS scale		PCOS (n=120)	Controls (n=143)	95%CI	OR	p
Depression Scale (HADS≥11)(%)		26 (21.7)	11 (7.7)	1.563 to 7.047	3.319	**0.001** 2
Anxiety Scale (HADS≥11)(%)		33 (27.5)	15 (10.5)	1.659 to 6.315	3.238	**0.001** 2

1 Independent-samples t-test.

2 Chi-square tests.

Categorical variables were shown as n% and continuous variables were shown as mean±SD or median (IQR) with regard to distribution characteristics. OR: odds ratio; BMI: body mass index; PCOS: polycystic ovary syndrome; HADS: Hospital Anxiety and Depression Scale. Bold indicates statistically significant p-values.

**Table 2:** Clinical and laboratory characteristics of women with polycystic ovary syndrome with depression (HADS≥11) and without depression (HADS<11).

	Depression (HADS≥11) (n=26)	Without depression (HADS<11) (n=94)	p
Age (years) median±IQR	27.0±9.0 (18–39)	25.0±6.0 (16–44)	0.745^1^
BMI (kg/m^2^) median±IQR	25.2±9.2 (17.4–38.9)	26.1± 7.3(17.9–45.8)	0.863^1^
Oligo/amenore (%)	19 (73.1)	64 (68.1)	0.626^2^
Irregular menstruation (%)	20 (76.9)	47 (50.0)	**0.001** 2
Ferriman-Gallwey score median±IQR	9.5±6.0 (5–18)	9.0 ± 5.0 (0–16)	**<0.014** 1
FSH median±IQR	5.6±(2.1) (3.4–9.8)	5.6±(1.7) (1.2–9.9)	0.977^1^
LH median±IQR	11.3±(7.5) (6.7–21.0)	7.2±(4.3) (1.4–21.0)	**<0.001** 1
LH:FSH median±IQR	2.3±(1.1) (0.7–5.5)	1.3±(0.8) (0.4–4.9)	**<0.001** 1
DHEA-SO_4_ median±IQR	209±(64.3) (70.4–357)	229.5±(99.3) (55.8–412)	0.424^1^
Prolactin median±IQR	15.2±(8.6) (7.6–45)	15.4±(11.8) (1.0–55.9)	0.990^1^
Total testosterone median±IQR	0.3±(0.2) (0.2–0.7)	0.3±(0.2) (0.1–0.9)	0.433^1^
TSH median±IQR	1.7±(1.2) (0.7–6.5)	1.8±(1.5) (0.3–5.5)	0.541^1^
Estradiol (E_2_) median±IQR	45.7±(20.5) (26–68)	45.9±(25.5) (11–170)	0.967^1^

1 Mann-Whitney U test.

2 Chi-square test.

BMI: body mass index; FSH: follicle-stimulating hormone; LH: follicle-stimulating hormone; LH/FSH: the ratio of luteinizing hormone to follicle-stimulating hormone; DHEA-SO_4_: dehydroepiandrosterone sulfate; TSH: thyroid-stimulating hormone; HADS: Hospital Anxiety and Depression Scale. Bold indicates statistically significant p-values.

**Table 3:** Clinical characteristics of women with polycystic ovary syndrome with anxiety (HADS≥11) and without anxiety (HADS<11).

	(Anxiety HADS≥11) (n=33)	Without anxiety (HADS<11) (n=87)	p
Age (years) median±IQR	27.0±9.0 (18–40)	25.0±5.0 (16–44)	0.128^1^
BMI (kg/m^2^) median±IQR	24.9±8.6 (17.4–35.9)	26.2±7.3 (17.9–45.8)	0.472^1^
Oligo/amenore (%)	25 (75.8)	58 (66.7)	0.336^2^
Irregular menstruation (%)	28 (84.8)	39 (44.8)	**<0.001** 2
Ferriman-Gallwey score median±IQR	9.0±6.0 (5–18)	7.0±5.0 (0–17)	**<0.001** 1
FSH median±IQR	5.7±(1.8) (2.2–9.9)	5.6±(1.8) (1.2–10)	0.892^1^
LH median±IQR	10.8±(7.3) (4.7–21)	7.3±(4.0) (1.4–17.0)	**0.001** 1
LH:FSH median±IQR	2.1±(1.0) (0.7–5.5)	1.3±(0.6) (0.4–3.5)	**0.003** 1
DHEA-SO4 median±IQR	230±(87.5) (70.4–412)	220±(87.0) (55.8–405)	0.711^1^
Prolactin median±IQR	18.6±(13.7) (7.6–55.6)	14.9±(10.8) (1.0–56.9)	0.109^1^
Total testosterone median±IQR	0.4±(0.3) (0.2–0.9)	0.3±(0.2)(0.1–0.7)	**0.040** 1
TSH median±IQR	1.8±(1.2) (0.7–4.7)	1.8±(1.5) (0.3–6.5)	0.981^1^
Estradiol (E_2_) median±IQR	44.0±(19.5) (26–106)	48.0±(25.3) (11–170)	0.486^1^

1 Mann-Whitney U test.

2 Chi-square test.

BMI: body mass index; FSH: follicle-stimulating hormone; LH: follicle-stimulating hormone; LH/FSH: the ratio of luteinizing hormone to follicle-stimulating hormone; DHEA-SO_4_: dehydroepiandrosterone sulfate; TSH: thyroid-stimulating hormone; HADS: Hospital Anxiety and Depression Scale. Bold indicates statistically significant p-values.

## DISCUSSION

This study showed that the prevalence of anxiety and depression behaviors was more common in women diagnosed with PCOS than in women without PCOS. When women with PCOS were evaluated within themselves, although there was no difference between the groups in terms of BMI, it was observed that menstrual irregularity and high Ferimann-Gallwey scores were associated with high depression and anxiety scores. Furthermore, blood LH levels, LH:FSH ratios, and total testosterone levels were higher in PCOS-diagnosed women with high anxiety scores.

Anxiety and depression are among the most common mental disorders^15,16^. Anxiety disorders are a group of mental disorders characterized by anxiety and fear, while depressive disorders are characterized by sadness, loss of interest and happiness, feelings of guilt and low self-esteem, and difficulty sleeping and concentrating^15^. In women, the global incidence of depression and anxiety is 5.1 and 4.6%, respectively. In addition, WHO data indicated that the prevalence of anxiety and depression in women aged 20–40 years was 5–6% and 6–7%, respectively^15^. Since the prevalence of anxiety and depression symptoms is high and the patients are benefitted from treatments very likely, routine screening was recommended^4^. In our study, the rates of high scores of anxiety and depression in all women with PCOS were 21.7 and 27.5%, respectively, and this result was similar to various studies that have reported a higher prevalence of anxiety and depression symptoms in women with PCOS than in healthy women^4^. Cooney et al., reported increased odds for depression and anxiety symptoms when the BMI was matched for PCOS and control groups^4^. Hollinrake et al., stated that obesity alone cannot explain the high risk of depression in PCOS^15^. Similarly, in this study, there was no difference in BMI values for the two study groups, and high depression and anxiety scores were recorded for the PCOS group. Irregular menstruation and hirsutism are common in women with PCOS, and some studies suggest that these features are associated with more severe anxiety and depression symptoms in women with PCOS^17,18^. Negative feelings about one's appearance, in particular, can contribute to poor mental health conditions such as anxiety and depression among women with PCOS^4^. Moreover, a meta-analysis by Cooney et al., reported that clinical and biochemical hyperandrogenism was associated with the prevalence of anxiety/depression-like behaviors in women with PCOS, suggesting that hirsutism, the clinical manifestation of hyperandrogenism, is associated with an increased prevalence of depression and anxiety^4^. In addition, Ekbäck et al., used the Ferriman-Gallwey score to evaluate hirsutism in their study and found that hirsutism was associated with anxiety and depression symptoms in women with PCOS^17^. In this study, Ferriman-Gallwey scores were significantly higher in the population with high scores of depression and anxiety. Despite numerous studies on this topic, it is still unclear which factors influence levels of anxiety and depression in women with PCOS. Therefore, the aim of our study was not only to determine the frequency of these disorders but also to evaluate the relationship of depression and anxiety with the hormone profile. Although serum LH and LH:FSH ratio were found to be statistically significant in women with PCOS with high anxiety and depression scale scores in our study, this finding was supported by many studies while others could not show this relationship^15,19,20^. In support of these studies, no difference was found in serum testosterone levels between women with PCOS with and without depression in our study. PCOS is a disease with hyperandrogenism, but depression and anxiety seem to be caused not by circulating androgen dominance, but by changes in body image like hirsutism. However, some studies show that exogenous DHEA-SO_4_ supplementation has beneficial effects on depression^20^.

Previous studies have also shown a relationship between high testosterone levels and anxiety^21^. In our study, although testosterone levels were similar in women with high depression scores, it was observed that testosterone levels were statistically significantly higher in women with PCOS with high anxiety scores. This result shows that the high anxiety score in women with PCOS may have an effect on biochemical hyperandrogenism as well as body image disorder.

One of the limitations of this study was its cross-sectional design, which limits our ability to assess the potential impact of PCOS on the future risk of depression and anxiety. Another limitation was a lack of data on a family history of mood disorders and potential confounding factors such as diet and physical activity.

Our work has several strengths. We confirmed the use of the Rotterdam criteria to diagnose PCOS in all participants and performed a preliminary assessment to confirm that the control group did not have undiagnosed PCOS.

In conclusion, it is clear that depression and anxiety are more common in patients with PCOS than in healthy women, and particularly, menstrual irregularities and hirsutism were related to depression and anxiety symptoms. Hyperandrogenism, which is one of the biochemical markers, appears to be associated with high anxiety scores and also causes hirsutism. The reason for this appears to be a cause–effect relationship between hirsutism and hyperandrogenism. Our findings support the previous recommendations about routine screening for depression and anxiety in this population. For managing PCOS, collaboration with nutritionists, endocrinologists, and behavioral medicine specialists might provide comprehensive care.

## Data Availability

The data that support the findings of this study are available from the corresponding author upon reasonable request.
